# Rostrum morphology and feeding strategy of the baleen whale indicate that right whales and pygmy right whales became skimmers independently

**DOI:** 10.1098/rsos.221353

**Published:** 2022-11-23

**Authors:** Yoshihiro Tanaka

**Affiliations:** ^1^ Osaka Museum of Natural History, Nagai Park 1-23, Higashi-Sumiyoshi-ku, Osaka 546-0034, Japan; ^2^ Hokkaido University Museum, Kita 10, Nishi 8, Kita-ku, Sapporo, Hokkaido 060-0810, Japan; ^3^ Numata Fossil Museum, 2-7-49, Minami 1, Numata town, Hokkaido 078-2225, Japan

**Keywords:** lunge feeding, skim feeding, Cetacea, Mammalia, convergent

## Abstract

Baleen whales have lost their functional teeth and begun to use their baleen plates to feed on small prey. Modern baleen whales exhibit different types of feeding strategies, such as lunging, skimming and so on. The evolution of feeding strategy in the Chaeomysticeti is an important step in considering niche partitioning and diversification, feeding efficiency and gigantism, and evolution and extinction. This study analyses the rostrum morphology to test the hypothesis that specific rostral morphologies facilitate special feeding strategies, using modern species and their observed feeding strategies. By this means, the convergence of rostral morphology can be recognized in the closest groups in the morphospace. As a result, the two linages (Balaenidae and *Caperea marginata*) are recognized to have convergent rostral morphology. In addition, an early member of the Chaeomysticeti, *Yamatocetus canaliculatus*, and most fossil species are plotted in or close to the cluster of lunge feeders. The original feeding strategy of the Chaeomysticeti could be more similar to lunge feeding than to skim feeding. Fossil relatives of the two linages showing transitional conditions indicate that they shifted to skim feeding independently. The evolution of the feeding strategy of the Chaeomysticeti is possibly more complex than that was thought.

## 1. Introduction

**Figure 1.**
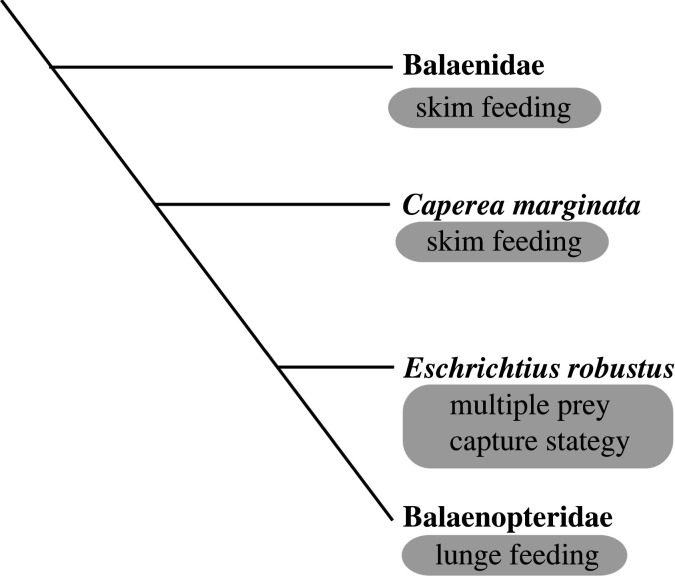
Modern baleen whale phylogeny and feeding strategies.

Baleen whales have lost their functional teeth and use their baleen plates to feed on zooplankton and small fish. Modern baleen whales exhibit three different types of feeding (figure 1 and table 1), such as skim feeding in balaenids and *Caperea marginata*, lunge feeding in most balaenopterids, and benthic suction in *Eschrichtius robustus*, and they have different combinations of feeding strategies. Lunge feeding is characterized by ‘intermittent engulfment and subsequent filtration’ [1]. Modern balaenopterids have throat grooves that expand to allow a huge volume of water intake, together with schools of prey [2]. On the other hand, skim feeding is characterized by ‘generating continuous negative pressure within the mouth cavity’ with a steady forward propulsion [3]. Skim feeders, such as balaenids, have a body that allows efficient cruising, but at slower speeds than those available to balaenopterids [4]. Interestingly, some species of mysticetes show a wider range of feeding methods. For example, *Balaenoptera borealis* can perform both skim and lunge feeding [5,6], which allows them to feed on smaller prey, in the first case, and larger prey or a greater density of prey, in the second [3].

**Table 1.** Known variation in feeding strategy among extant mysticetes. See cited references in the electronic supplementary material, file S1.

scientific name	feeding style	reference
*Balaena mysticetus*	skim feeding	Pivorunas, 1979; Nemoto, 1970
*Eubalaena japonica*	skim feeding	Pivorunas, 1979; Nemoto, 1970
*Eubalaena glacialis*	skim feeding	Pivorunas, 1979; Nemoto, 1970
*Caperea marginata*	skim feeding	Pivorunas, 1979; Nemoto, 1970
*Eschrichtius robustus*	multiple prey capture strategy. Benthic lateral suction (Scammon, 1874; Kasuya and Rice, 1970; Pivorunas, 1979) skim feeding and gulp (Nemoto, 1970; Jefferson *et al.,* 2008) capable of lunge feeding (Werth, 2000)	Scammon, 1874; Kasuya and Rice, 1970; Pivorunas, 1979; Nemoto, 1970
*Megaptera novaeangliae*	lunge feeding, bottom feeding (Hain *et al*., 1995)	Pivorunas, 1979; Jurasz and Jurasz, 1979; Frisch-Jordan *et al.,* 2019
*Balaenoptera acutorostrata*	lunge feeding	Pivorunas, 1979
*Balaenoptera bonaerensis*	lunge feeding	Pivorunas, 1979
*Balaenoptera edeni*	lunge feeding	Pivorunas, 1979; Iwata *et al.*, 2017
*Balaenoptera brydei*	lunge feeding	Pivorunas, 1979
*Balaenoptera borealis*	multiple prey capture strategy. Skim feeding for smaller, and lunge feeding (lunging) for larger or greater density of prey (Brodie and Vikingsson, 2009)	Ingebrigtsen, 1929; Pivorunas, 1979; Nemoto, 1959, 1970; Brodie, 1975; Brodie and Vikingsson, 2009; Horwood, 2018; Segre *et al*., 2021
*Balaenoptera physalus*	lunge feeding	Pivorunas, 1979
*Balaenoptera musculus*	lunge feeding	Pivorunas, 1979
*Balaenoptera omurai*	lunge feeding	Jefferson, 2008
*Balaenoptera ricei*	unknown	unknown (Rosel *et al*., 2021); bottom or near-bottom feeding? (Soldevilla *et al*., 2017)

The evolution of the Mysticeti feeding strategy in the toothed baleen whale has been well studied recently, using the morphology of the tooth, skull and mandible. Before baleen-assisted filter feeding was evolved, toothed mysticetes employed variable feeding strategies, such as suction, suction-assisted filter and suction-assisted raptorial feeding [7–14].

On the other hand, later mysticetes, such as true baleen whales (the Chaeomysticeti), have been investigated, but not in the way that toothed baleen whales have. The Chaeomysticeti are a group of toothless mysticetes containing all extant baleen whales. Identifying the evolution of feeding strategies of the Chaeomysticeti will be an important step in considering niche partitioning and diversification, feeding efficiency and gigantism, and evolution and extinction in detail.

Several recent studies have described the original feeding strategy of the Chaeomysticeti. An early review paper on feeding mechanism of the Mysticeti noted that fossil mysticetes were structurally similar to balaenopterids and *Eschrichtius robustus* [15]. A later review more clearly showed that lunge feeding was the strategy used by archaic baleen whales because none of them display arched rostra like the Balaenidae or a robust rostrum like *Eschrichtius robustus* [16]. This view is compatible with the result of a later study, which suggested that the early Chaeomysticeti *Toipahautea waitaki* was considered as a possible gulp-feeder, based on the mandible structures [17]. Later, a study of injuries to fossil mysticetes reported that osteosclerotic ribs can be seen in primitive mysticetes [18]. These ribs suggest that the earliest Chaeomysticeti employed benthic feeding.

Recently, the Eomysticetidae, an early group of the Chaeomysticeti, has come to be considered a skim feeder based on its lack of lunge feeder features, such as having a delicate temporomandibular joint, non-laterally deflected coronoid process of the mandible and anteroposteriorly expanded rostrum [19]. The study also emphasized that members of the Eomysticetidae are skim feeders like the Balaenidae ‘as the next diverging lineage of mysticetes suggests that skim feeding may reflect the primitive mode of feeding among the Chaeomysticeti’ (figure 1). These informative and frontier studies enable the development of hypotheses and have increased interest in the evolution of feeding strategy among the Chaeomysticeti.

Modern baleen whale feeding behaviours have been observed directly [20] and through examination of gut contents [5]. However, there are many soft tissues, such as the expandable ventral pouch and the synovial craniomandibular joint that can be seen in the modern baleen whales but not in fossils [21]. In addition, fossil specimens are rare, incomplete and are deposited in institutions globally. These facts make it difficult to access them to take photos or measurements and to examine specimens directly [22].

In the head, the rostrum, palate, temporomandibular joint and teeth/baleen in particular are associated with feeding strategy [14]. Previous studies have focused on the mandible, especially in the Cetotheriidae and Balaenopteridae [23–30]. As noted above, previous studies have sought to identify the early feeding strategy of the Chaeomysticeti, but have not provided a final assessment [31]. The rostrum seems to be an important element to consider with respect to feeding strategy, but it is easily detached and not commonly preserved in fossils.

The objective of this study is to add additional data to take into account the early feeding strategy of the Chaeomysticeti, using a poorly analysed but possibly closely related element, the rostrum. The hypothesis that specific rostral morphologies facilitate specific feeding strategies can be tested using known modern baleen whale feeding strategy. Then, comparing the positions of earlier chaeomysticetes in the morphospace of the rostrum provides polarity of feeding strategy evolution. Finally, the recognition of the convergence of the rostral morphology of extant species can be used to support the hypothesis.

## 2. Materials and methods

**Figure 2.**
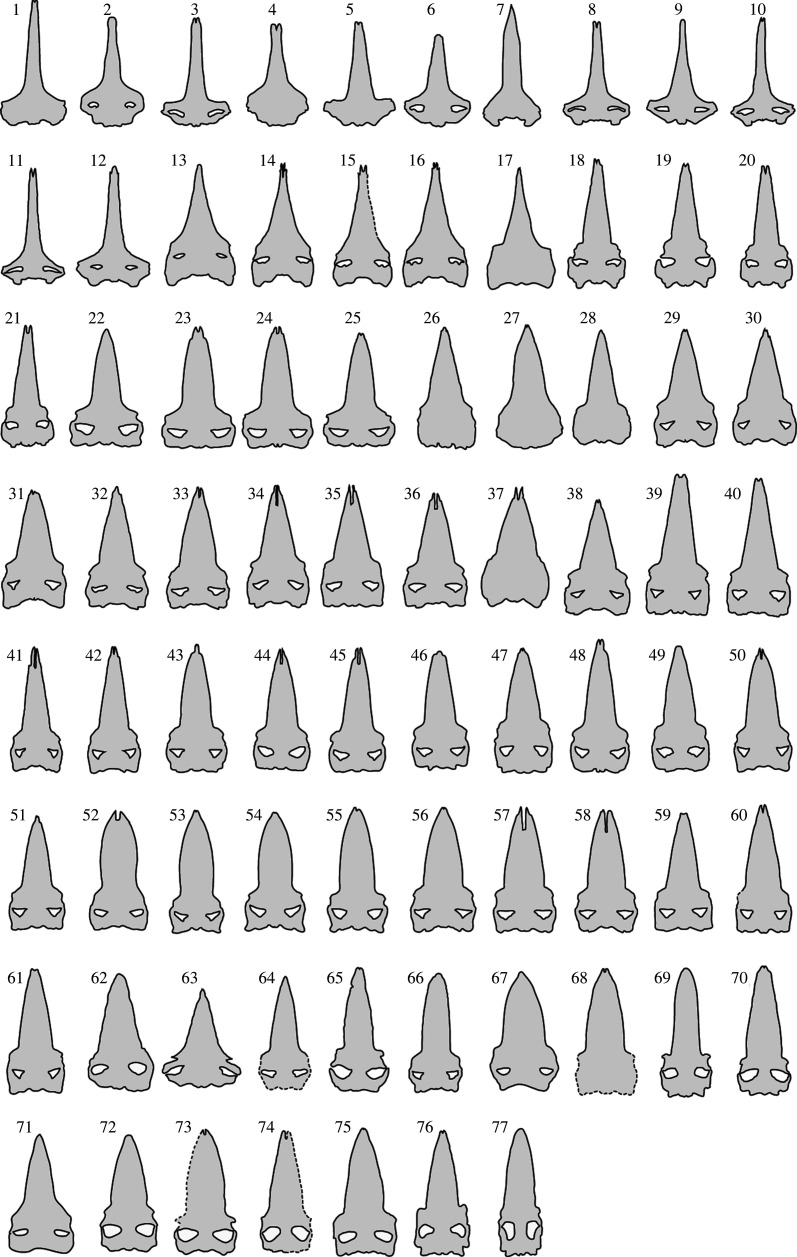
Outline of analysed true baleen whale specimens. Numbers are given in table 2 and the electronic supplementary material, file S2.

The anatomical terms used here follow Mead & Fordyce [32]. Skull data were collected from 77 specimens seen in previous studies (figure 2 and table 2; see also the electronic supplementary material, file S2). They include 16 extant species of 61 specimens representing all extant baleen whales. Extinct toothless baleen whales (members of the Chaeomysticeti) were selected through the preservation of their rostrum. The rostrum is a combination of thin bones. Some specimens were reconstructed using a preserved left or right side of the specimens.

**Table 2.** Specimens that were used for analyses in this study. See cited references in the electronic supplementary material, file S2.

family	scientific name	Specimen number or ID	ID	Reference
Balaenidae	*Balaena mysticetus*	—	1	Nishiwaki and Kasuya, 1970
	*Eubalaena australis*	Table XXV, fig. 5	2	Cuvier, 1823
	*Eubalaena australis*	Table XXV, fig. 7	3	Cuvier, 1823
	*Eubalaena australis*	—	4	Van Beneden and Gervais, 1868
	*Eubalaena australis*	USNM 267612	5	Best, 2008
	*Eubalaena australis*	—	6	Jefferson *et al*., 1999
	*Eubalaena glacialis*	Table XXV, fig. 11	7	Cuvier, 1823
	*Eubalaena glacialis*	USNM 23077	8	True, 1904
	*Eubalaena glacialis*	—	9	Allen, 1908
	*Eubalaena japonica*	61A	10	Omura, 1969
	*Eubalaena japonica*	61B	11	Omura, 1969
	*Eubalaena japonica*	—	12	Omura, 1958
Neobalaenidae	*Caperea marginata*	—	13	Jefferson *et al*., 1999
	*Caperea marginata*	ZM 39768	14	Best, 2008
	*Caperea marginata*	OM VT227	15	ー
	*Caperea marginata*	MM002235	16	Fordyce and Marx, 2012
	*Caperea marginata*	—	17	Beddard, 1901
Eschrichtiidae	*Eschrichtius robustus*	M-804A	18	Nakamura and Kato, 2014
	*Eschrichtius robustus*	M-804B	19	Nakamura and Kato, 2014
	*Eschrichtius robustus*	AMNH 34260	20	Andrews, 1914
	*Eschrichtius robustus*	USNM A13803	21	Andrews, 1914
Balaenopteridae	*Megaptera novaeangliae*	USNM 269982	22	Best, 2008
	*Megaptera novaeangliae*	USNM 21492	23	True, 1904
	*Megaptera novaeangliae*	USNM 16252/13656	24	True, 1904
	*Megaptera novaeangliae*	Milwankee Public Museum	25	True, 1904
	*Megaptera novaeangliae*		26	Van Beneden and Gervais, 1868
	*Megaptera novaeangliae*		27	Cope, 1871
	*Balaenoptera acutorostrata*	—	28	Van Beneden and Gervais, 1868
	*Balaenoptera acutorostrata*	ZM 41590	29	Best, 2008
	*Balaenoptera acutorostrata*	NMNS M42450	30	Marx *et al*., 2016
	*Balaenoptera acutorostrata*	—	31	Arnold *et al*., 1987
	*Balaenoptera bonaerensis*	71J2793	32	Omura, 1975
	*Balaenoptera bonaerensis*	71J2883	33	Omura, 1975
	*Balaenoptera acutorostrata*	AY69B	34	Omura, 1975
	*Balaenoptera acutorostrata*	USNM 20931	35	True, 1904
	*Balaenoptera acutorostrata*	USNM 13877	36	True, 1904
	*Balaenoptera acutorostrata*	NFL	37	—
	*Balaenoptera bonaerensis*	ZM 39861	38	Best, 2008
	*Balaenoptera borealis*	—	39	Van Beneden and Gervais, 1868
	*Balaenoptera borealis*	USNM 504244	40	Best, 2008
	*Balaenoptera borealis*	—	41	Nishiwaki and Kasuya, 1971
	*Balaenoptera borealis*	AMNH 34871	42	Andrews, 1916
	*Balaenoptera brydei*	TN9903	43	Yamada *et al*., 2006
	*Balaenoptera brydei*	—	44	Omura, 1959
	*Balaenoptera edeni*	77N62, Plate 1	45	Omura *et al*., 1981
	*Balaenoptera edeni*	78N33, Plate 2	46	Omura *et al*., 1981
	*Balaenoptera edeni*	KINMEN01	47	Yamada *et al*., 2006
	*Balaenoptera edeni*	—	48	Jefferson *et al*., 1999
	*Balaenoptera edeni*	—	49	Junge, 1950
	*Balaenoptera ricei*	USNM 594665	50	Rosel *et al*., 2021
	*Balaenoptera ricei*	USNM 572922	51	Rosel *et al*., 2021; Best, 2008
	*Balaenoptera musculus*	—	52	Van Beneden and Gervais, 1868
	*Balaenoptera musculus*	—	53	Jefferson *et al.*, 1999
	*Balaenoptera musculus*	USNM 124326	54	Best, 2008
	*Balaenoptera musculus brevicauda*	—	55	Omura, 1970
	*Balaenoptera omurai*	NMNS M32505	56	Wada *et al.*, 2003
	*Balaenoptera omurai*	PMBC11621	57	Yamada *et al*., 2006
	*Balaenoptera omurai*	SAM M21245	58	Yamada, Kemper *et al.*, 2006
	*Balaenoptera physalus*	USNM 237566	59	Best, 2008
	*Balaenoptera physalus*	Philadelphia Academy of Natural Science	60	True, 1904
	*Balaenoptera physalus*	USNM 16039	61	True, 1904
extinct taxa				
stem Balaenopteroidea or Cetotheriidae	*Titanocetus sammarinensis*	MGB1CMC1729073	62	Bisconti, 2006
Balaenidae	*Balaenella brachyrhynus*	NMB 42001	63	Bisconti, 2005
Balaenopteridae	*Archaebalaenoptera castriarquati*	SBAER 240536	64	Bisconti, 2007a
Balaenopteridae	*Protororqualus cuvieri*	—	65	Bisconti, 2007b
Balaenopteridae	*Incakujira anillodefuego*	GNHM Fs-098-12	66	Marx and Kohno, 2016
Balaenopteridae	*Balaenoptera siberi*	—	67	Pilleri, 1989
Balaenopteridae	*Plesiobalaenoptera quarantellii*	MPST 240505	68	Bisconti, 2010
Cetotheriidae	*Piscobalaena nana*	NMNH SAS1617	69	Marx *et al.*, 2017
Cetotheriidae	*Diorocetus hiatus*	USNM 16783	70	Kellogg, 1968
Neobalaenidae	*Miocaperea pulchra*	SMNS 46978	71	Marx and Fordyce, 2016
*Isanacetus* and related clade	*Isanacetus laticephalus*	MFM 28501	72	Kimura and Ozawa, 2002
*Isanacetus* and related clade	*Pelocetus calvertensis*	USNM 11976	73	Kellogg, 1965
*Isanacetus* and related clade	*Parietobalaena palmeri*	USNM 10677	74	Kellogg, 1968
*Isanacetus* and related clade	*Atlanticetus patulus*	USNM 23690	75	Kellogg, 1968
*Isanacetus* and related clade	*Mixocetus elysius*	LACM 882	76	Kellogg, 1934
Eomysticetidae	*Yamatocetus canaliculatus*	KMNH VP 000,017	77	Okazaki, 2012

### 2.1. Institutional abbreviations

AMNH, American Museum of Natural History, New York, USA. GNHM, Gamagori Natural History Museum, Japan. KMNH, Kitakyushu Museum of Natural History, Fukuoka, Japan. LACM, Natural History Museum of Los Angeles County, Los Angeles, USA. MFM, Mizunami Fossil Museum, Gifu, Japan. MGB, Museo Geopalaeontologico G. Capellini, Bologna, Italy. MNHN, Muséum National d'Histoire Naturelle, Paris, France. MPST, Museo Paleontologico di Salsomaggiore Terme, Italy. MUSM, Museo de Historia Natural, Universidad Nacional Mayor de San Marco, Lima, Peru. NMB, Natuurmuseum Brabant, Tilburg, The Netherlands. NMNS, National Museum of Nature and Science, Tsukuba, Japan. PMBC, Phuket Marine Biological Center, Puket, Thailand. SAM, South Australian Museum, Adelaide, Australia. SBAER, Soprintendenza per i Beni Archeologici dell’ Emilia Romagna. SBAER, Soprintendenza per i Beni Archeologici dell'Emilia Romagna, Italy. SMNS, Staatliches Museum für Naturkunde, Stuttgart, Germany. USNM, National Museum of Natural History, Smithsonian Institution, Washington, DC, USA. ZM, IZIKO South African Museum, Cape Town, South Africa.

### 2.2. Data collection

**Figure 3.**
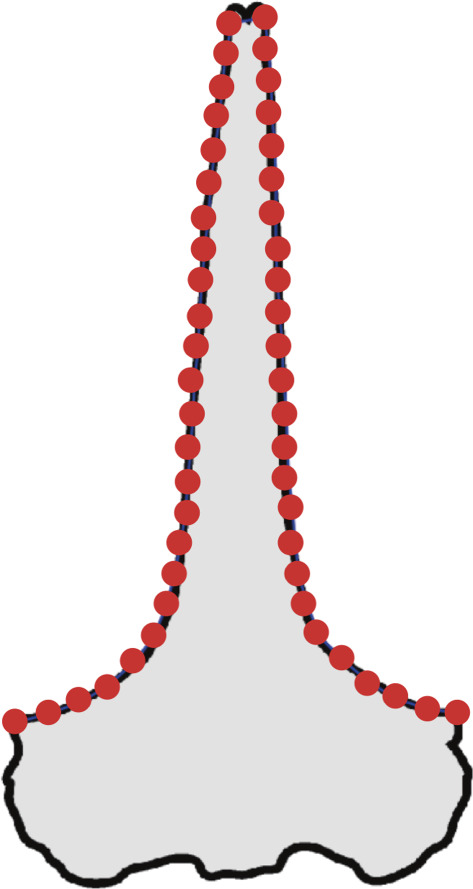
Example semi-landmark in the ventral view of *Balaena mysticetus* skull.

Landmark acquisition was managed using the TPS program package, including tpsUtil v1.78 and tpsDig v2.31 [33]. Semi-landmarks (figure 3) were measured on each specimen. Lines on the margin of the skull were taken as semi-landmarks between right and left anterolateral ends of the rostrum on pictures in dorsoventral view. They were divided into 50 semi-landmarks at equal distances. Non-shape information (size and rotation) was removed from the landmark configurations using the New Procrustes Fit implemented in MorphoJ 1.07a [34].

### 2.3. Morphometric analysis

Geometric morphometric analysis was used to access the shape variation of the rostrum morphology and test the hypothesis that specific rostral morphologies facilitate specific feeding strategies. All analyses were run using MorphoJ 1.07a [34].

Principal component analysis (PCA) was used to reduce the dimensionality of the data, and to display the major axes of variation for extant and extinct true baleen whales [34]. In the analysis, PCA was used to identify the positions of earlier chaeomysticetes in the morphospace with the modern species (figure 5) and to recognize the convergence of the rostral morphology of extant species categorized by linages (figure 6). If specific rostral morphologies facilitate specific feeding strategies, then clusters of phylogenetically separated linages with the same feeding strategies will be closely associated.

The feeding strategies of recently established extant species *Balaenoptera ricei* and extinct true baleen whales were assigned as unknown (not observed) [35]. Interestingly, *B. ricei* dives deep and forages at or near the sea floor during the day [36], which is an unusual feeding strategy of balaenopterids.

### 2.4. Cladograms

To consider the evolution of feeding strategies among the Chaeomysticeti, the estimated feeding strategies of extinct true baleen whales from PCA (figure 6 and table 3) are adapted to match previous phylogenetic hypotheses. The feeding strategies among the Chaeomysticeti have been shifted from certain primitive feeding strategies to modern baleen whales, which have variable feeding strategies. Here previous phylogenetic hypotheses are combined with the results to recognize the polarity in feeding strategy.

**Table 3.** Estimated feeding strategies for extinct taxa and *Balaenoptera ricei*.

ID	family	scientific name	close feeding strategy of extant whales	estimated feeding strategy
50, 51	Balaenopteridae	*Balaenoptera ricei*	lunge feeding	lunge feeding
62	stem Chaeomystideti	*Titanocetus sammarinensis*	lunge feeding	a primitive feeding
63	Balaenidae	*Balaenella brachyrhynus*	more or less the same distances from lunge and skim feeders	transitional feeding strategy from a primitive one to skim feeding
64	Balaenopteridae	*Archaebalaenoptera castriarquati*	lunge feeding	a kind of lunge feeding
65	Balaenopteridae	*Protororqualus cuvieri*	lunge feeding	a kind of lunge feeding
66	Balaenopteridae	*Incakujira anillodefuego*	lunge feeding	a kind of lunge feeding
67	Balaenopteridae	*Balaenoptera siberi*	lunge feeding	a kind of lunge feeding
68	Balaenopteridae	*Plesiobalaenoptera quarantellii*	lunge feeding	a kind of lunge feeding
69	Cetotheriidae	*Piscobalaena nana*	more or less the same distances from lunge and multiple prey capture strategies	possibly could do some feeding ways
70	Cetotheriidae	*Diorocetus hiatus*	lunge feeding	a primitive feeding
71	Neobalaenidae	*Miocaperea pulchra*	lunge feeding	a primitive feeding
72	*Isanacetus* and related clade	*Isanacetus laticephalus*	lunge feeding	a primitive feeding
73	*Isanacetus* and related clade	*Pelocetus calvertensis*	lunge feeding	a primitive feeding
74	*Isanacetus* and related clade	*Parietobalaena palmeri*	lunge feeding	a primitive feeding
75	*Isanacetus* and related clade	*Atlanticetus patulus*	lunge feeding	a primitive feeding
76	*Isanacetus* and related clade	*Mixocetus elysius*	lunge feeding	a primitive feeding
77	Eomysticetidae	*Yamatocetus canaliculatus*	lunge feeding	a primitive feeding

Numerous phylogenetic hypotheses for baleen whales exist, and some show a clade of the Balaenidae and *Caperea marginata* (e.g. [19,30]), which is not supported by molecular phylogenetic analyses. Ones of trees are confluent with phylogenetic relationships of the Balaenidae and *Caperea marginata* based on molecular data [37–40]; these are used in this study. These hypotheses do not reach consensus on contents of the Cetotheriidae, branching patterns of the Balaenopteridae, Cetotheriidae and other so-called cetotheres, and the position of some key basal taxa (*Titanocetus sammarinensis, Aglaocetus moreni* and *Atlanticetus patulus*). Such phylogenetic hypotheses can be recognized in two types in this study. The two patterns differ in their placement of so-called cetotheres in the crown group (Type A [41–47]) or placing many ‘cetotheres’ basal to the Balaenidae (Type B [7,48]).

## 3. Results

### 3.1. Principal component analysis

The first two PCs combined explain 70.0% of the variation (PC1 = 50.6%, PC2 = 19.4%, PC3 = 17.8%, PC4 = 6.4%, PC5 = 1.7%, PC6 = 0.9%), and the results of Procrustes ANOVA in the shape of feeding strategies were significant (*p* < 0.001) (electronic supplementary material, file S3).

**Figure 4.**
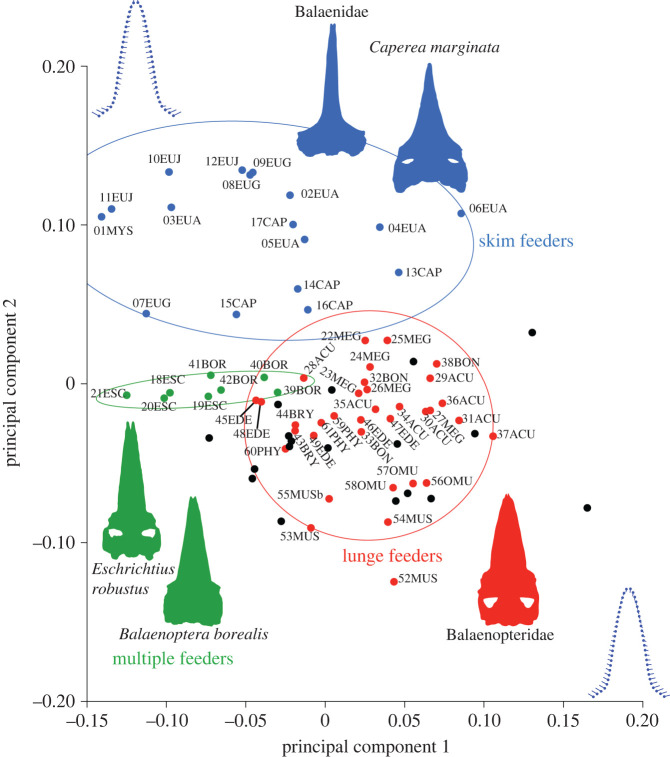
The results of PCA showing the IDs of modern taxa. Ovals represent 90% confidence intervals for each distinctive feeding strategy of the extant taxa. Diagrams of the shape changes in the positive directions are given along each axis. Numbers and letters are IDs and abbreviations of scientific names (table 2).

Principal component 1 represents a contrast of the lateral margin at the anteroposterior middle level of the rostrum and relative length of the rostrum. To the right end (the positive side) of PC1, the rostrum is wider, has swollen lateral margins and is shorter. By contrast, to the left (the negative side), the rostrum is slender and its lateral margins are straight (figure 4). *Eschrichtius robustus* and *Balaenoptera borealis* as multiple prey capture feeders have negative PC1 scores associated with straight rostra and narrow bases. Lunge feeders (most of balaenopterids) and some skim feeders (*Eubalaena australis* and *Caperea marginata*) have near-zero to positive PC1 scores associated with wide and short rostra. Most fossil taxa have near-zero to positive PC1 scores and are most similar in rostrum morphospace to the lunge feeders (balaenopterids but *B. borealis*).

Principal component 2 is characterized by changes in the narrowness of the rostra. Positive PC2 scores were related to a wide base and sharp rostrum that can be seen in skim feeders (Balaenidae and *Caperea marginata*). *Eschrichtius robustus* (a benthic suction + skim + lunge feeder) and *Balaenoptera borealis* (a skim + lunge feeder) have near-zero PC2 scores. Negative PC2 scores were associated with decreased sharpness of rostra, as can be seen in some lunge feeders (*Balaenoptera musculus* and *B. omurai*).

**Figure 5.**
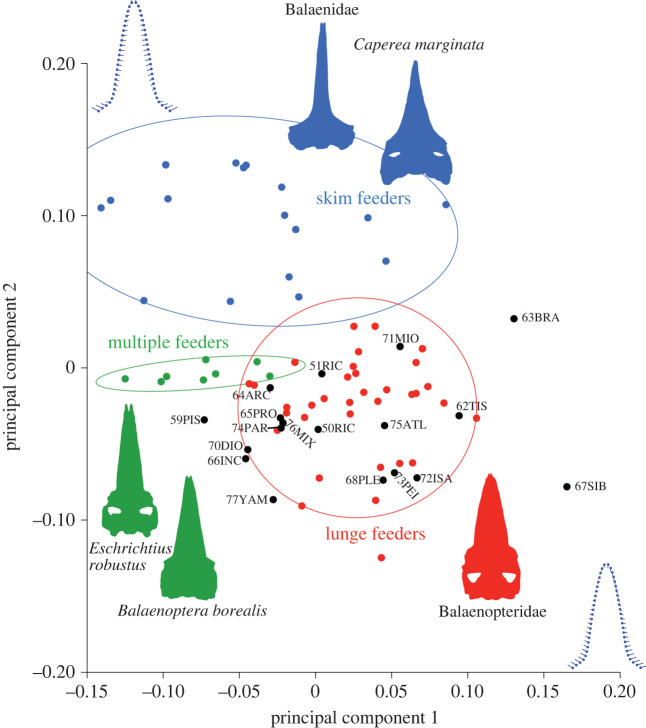
The same results as in figure 4 showing IDs of fossils and *Balaenoptera ricei*, which are unknown in feeding strategies. Ovals represent 90% confidence intervals for each distinctive feeding strategy of extant taxa. Diagrams of the shape changes in the positive directions are shown along each axis. Numbers and letters are IDs and abbreviations of scientific names (table 2).

Fossil taxa are most closely associated with the cluster of the Balaenopteridae made up of lunge feeders in the morphospace (figure 5). Most importantly, the early Chaeomysticeti *Yamatocetus canaliculatus* shows a negative PC2 score, in the context that most balaenopterids are scored negative PC2, which make *Yamatocetus canaliculatus* closest to the clusters of the Balaenopteridae instead of skimmers (the Balaenidae and *Caperea marginata*).

Three fossil taxa are plotted far from all extant species such as *Balaenella brachyrhynus, Piscobalaena nana* and *Balaenoptera siberi*. *Balaenella brachyrhynus* is plotted close to the 90% confidence ellipse for skim feeders and outside of 90% confidence ellipses of both skim and lunge feeders. *Piscobalaena nana* is plotted equally distant from the lunge feeders (balaenopterids) and *Eschrichtius robustus* and *Balaenoptera borealis,* which exhibit a multiple prey capture strategy. *Balaenoptera siberi* was plotted far from the others, but the cluster of lunge feeders is the closest of all clusters. In addition, *Diorocetus hiatus* was plotted near the lunge feeder cluster.

The newly described modern species *Balaenoptera ricei* is supposed to be a lunge feeder as it was plotted near the centre of the lunge feeders' cluster. More reports on this mysterious whale’s field research will help to identify the truth.

Two linages of skimmers, the Balaenidae and *Caperea marginata*, are most closely associated (figure 4). Two linages of multiple prey capture feeders, *Eschrichtius robustus* and *Balaenoptera borealis*, are most closely associated.

## 4. Discussion

### 4.1. Earlier feeding strategy of the Chaeomysticeti was not skimming

*Yamatocetus canaliculatus* from the early Oligocene (about 29–28 Ma) is an early member of the Eomysticetidae, which is the most basal family among the Chaeomysticeti, the true baleen whales [9,49].

Importantly, not only *Yamatocetus canaliculatus* (number 77 in figure 5), but also most of the analysed fossil taxa are plotted in or close to the cluster of lunge feeders, instead of among skim and multiple prey capture strategy clusters in the analysis, as noted in the Results section. This result indicates that the earlier feeding strategy of the Chaeomysticeti in earlier times was not skimming, as it is in modern balaenids and *Caperea marginata*.

The holotype of *Eomysticetus whimorei* preserving the anterolateral borders of the rostrum is not included in the analyses because of their limited preservation, but is similar to the rostrum of *Yamatocetus canaliculatus* in its proportions [50]. It is highly possible that the rostrum of *Yamatocetus canaliculatus* represents the rostrum shape of the Eomysticetidae.

The early chaeomysticetes, *Sitsqwayk cornishorum* and *Tokarahia kauaeroa*, are key taxa, but their holotypes do not preserve the rostrum. Another key early Chaeomysticeti, *Toipahautea waitaki,* also cannot be included in these analyses because of the limited preservations of the rostrum, but it appearss to have had a wide posterior part to its rostrum, unlike those of modern balaenids. This species is considered to be a possible lunge feeder, based on its mandible structures [17].

In addition, another early member of the Chaeomysticeti, *Horopeta umarere,* was considered a lunge feeder based on the presence of features shared with the Balaenopteridae lunge feeders, such as a laterally bowed robust mandible and a posterolaterally deflected triangular coronoid process [51]. These taxa indicate that the feeding strategy of early or Oligocene Chaeomysticeti was not skim feeding, and it may have been a primitive version of lunge feeding.

Eomysticetidae are thought of as skim feeders because of their lack of balaenopterid mandible features and phylogenetic branching patterns of the modern skimmers Balaenidae and Eomysticetidae (figure 1) [19]. Indeed, elimination is a powerful logical thinking tool. However, we may not be able to use elimination for all cases, such as postulating the feeding strategies of the past whales, because some feeding strategies might have vanished. If some feeding strategies exist and do not survive to the present, they do not allow us to develop a complete set of feeding strategies to eliminate.

In this study, one possibility is eliminated (skim feeding), but the primitive feeding strategy cannot be determined from among the feeding strategies of modern baleen whales because the real strategy might not exist in the present. However, the analysis indicates that the feeding strategy of the Chaeomysticeti was close to lunge feeding, but was not the same as the lunge feeding employed by modern balaenopterids. Thus, the fossil taxa are considered to be a kind of primitive feeder (table 3).

### 4.2. Appearance and shift of the two skim feeder linages through Chaeomysticeti evolution

The results suggest that specific rostral morphologies facilitate specific feeding strategies among modern mysticetes (figure 5). Skim feeders show slender rostra with medially excavated lateral borders of the rostrum. Baleen whales using a multi-prey capture strategy show a straight and moderate width of the rostrum. Lunge feeders show wide rostra with laterally expanded borders.

The most strongly supported specific rostral morphology and feeding strategy is that of skim feeders, as the Balaenidae and *Caperea marginata* show convergent evolution. Using molecular phylogenetic analyses, *Caperea marginata* forms a clade with the Balaenopteridae instead of the Balaenidae [37–40]. The Balaenidae and *C. marginata* are not a monophyletic group, but the two linages share rostrum features and a feeding strategy gained through convergent evolution.

The rostra of the Balaenidae and *Caperea marginata* are medially excavated in dorsoventral views, anteriorly narrow and posteriorly dramatically wide. In addition, they share a long and open palatal maxillary sulci, short zygomatic processes and atrophied coronoid process, which differ from those of the Balaenopteridae [52,53]. These features are also probably convergent across the two groups, and their palatal maxillary sulci are maintained with much longer baleen plates than those of balaenopterids.

Having medially excavated lateral borders of the rostrum could be related to having cross-flow filtering during skim feeding. Previously, balaenids were thought to do skim feeding by dead-end filtering [54]. Currently, we know that cross-flow filtering is the way for balaenids [54–56]. One advantage of cross-flow filtering is minimizing clogging, as the fluid is filtered by the flow parallel to the filter surface [57]. Holding a large filtering surface inside of the mouth and a smaller anterior entrance to the mouth is an advantage of using cross-flow filtering for the Balaenidae and *Caperea marginata*, with their long baleen plates. The fluid dynamics of the Balaenidae skim feeding can be described as follows [56]. Water including particles enters from the anterior tip of the mouth, which features an opening due to the lack of the baleen plates. The fluid flows transversely between the baleen plates. This means that a water orientation from anterior to posterior naturally moves cross-flow against the series of the baleen plates, which allows them to have their large (dorsoventrally high and anteroposteriorly long) filtering surface.

Why are the lateral borders excavated medially? Their excavation is not related to the distribution of baleen plate, as the palatal maxillary sulci showing the base of the baleen plates are distributed in a straight pattern, not a curved one, following the lateral borders of the rostrum. Thus, a reason for having the medially excavated lateral borders would be to fill the gap between the very narrow anterior part and the much wider posterior sensory structures such as the orbit and crania. It seems likely that, from a hydrodynamic point of view, the gap is better filled with a stream shape line.

**Figure 6.**
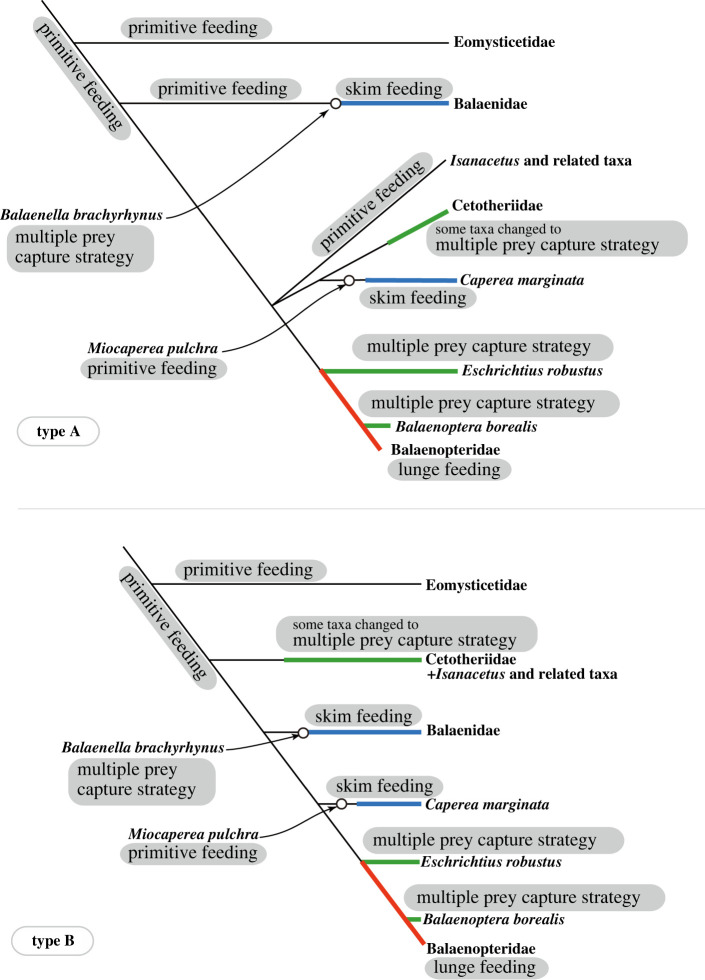
Feeding strategy evolution among the Chaeomysticeti: true baleen whales with two types of phylogenetic hypotheses. Thin green lines indicate linages with unknown primitive feedings. Thick lines represent shifts from primitive feeding to skim feeding in blue, to multiple prey capture strategy in green, and lunge feeding as seen among modern balaenopterids in red.

Here, the evolutionary history of the feeding strategy of the Chaeomysticeti is partially described, given with many limits. We still do not have a clear idea of the early feeding strategy of early chaeomysticetes, but this did not involve skim feeding, and it might be close to lunge feeding, as discussed above. Previous phylogenies have used molecular data and estimated feeding strategy polarity as above to consider evolution of the feeding strategy among the Chaeomysticeti. As noted in the methodology section, two types of phylogenetic hypotheses are used to consider feeding strategy evolution among true baleen whales. Both types of phylogenetic hypotheses support more or less the same trends of the feeding stage of evolution of the Chaeomysticeti (figure 6).

In the feeding strategy history of the Chaeomysticeti, skim feeders independently appeared at least twice in the Balaenidae and *Caperea marginata* linages from some sort of primitive feeders. This hypothesis is supported by fossil relatives of the two linages (figure 6).

*Miocaperea pulchra* is a fossil relative of *Caperea marginata* [58]. In this study, *Miocaperea pulchra* (number 71 in figure 5) is plotted in the clusters of lunge feeders, and its position is close to the cluster of skim feeders. It can be estimated as a transitional feeder from a primitive to skim feeding (table 3). The species shows laterally slightly expanded lateral borders of the rostrum.

The feeding strategy of *Balaenella brachyrhynus,* a fossil taxon of the Balaenidae, was unknown, because of the lack of complete mandibles [59]. However, feeding strategies of Pliocene balaenids were thought to be different from those of modern balaenids due to their different conditions of the skull and mandible [60,61]. In this study, *Balaenella brachyrhynus* (number 63 in figure 5) is plotted at more or less the same distances from the clusters of lunge and skim feeders, and a moderate rostrum condition is shown between the clusters of the lunge and skim feeders. These facts imply that the Balaenidae and *Caperea* linages changed their feeding strategy from primitive feeding, which is considered to have been similar to lunge feeding, to skim feeding through moderate rostrum morphologies.

## 5. Conclusion

This study examined the relationships of the rostrum shape among fossils and modern baleen whales and recognized convergent evolution of the feeding strategy and rostral morphology in modern baleen whales. As a result of analyses, the most basal family Eomysticetidae and most fossil taxa were plotted in or close to the cluster of the lunge feeders. This eliminated the possibility that skim feeding in the way that modern balaenids and *Caperea marginata* do is not an adequate feeding strategy of the early Chaeomysticeti. Skim feeders have slender rostra with a medially excavated lateral border of the rostrum. On the other hand, lunge feeders show very wide rostra. The result suggested convergent evolution of skim feeder rostrum, which is slender and medially excavated. These results imply that two linages (the Balaenidae and *Caperea marginata*) shifted from primitive to skim feeding independently.

Because the lunge feeders are a monophyletic group, we cannot recognize convergence of lunge feeding among the modern baleen whales. Thus, although most fossil chaeomysticetes are plotted near to the centre of the cluster of lunge feeders, they nevertheless cannot be recognized as lunge feeders, which is a limitation of this study. The lunge feeding of modern balaenopterids is among the most specialized feeding strategies, and it is employed by many derived anatomical features. Thus, this specialized condition of the feeding strategy is not likely to resemble the primitive feeding of the Chaeomysticeti. To determine how primitive feeding strategies worked, data on other anatomical features are needed.

## Data Availability

The data are provided in the electronic supplementary material [62].

## References

[1] Croll DA, Tershy BR, Newton KM, de Vos A, Hazen E, Goldbogen JA. 2018 Filter feeding. In Encyclopedia of marine mammals, pp. 363-368. Amsterdam, The Netherlands: Elsevier.

[2] Nemoto T. 1959 Food of baleen whales with reference to whale movements. Sci. Rep. Whales Res. Inst. Tokyo **14**, 149-291.

[3] Brodie P, Vikingsson G. 2009 On the feeding mechanisms of the sei whale (*Balaenoptera borealis*). J. Northwest Atlantic Fish. Sci. **42**, 49-54.

[4] Woodward BL, Winn JP, Fish FE. 2006 Morphological specializations of baleen whales associated with hydrodynamic performance and ecological niche. J. Morphol. **267**, 1284-1294. (10.1002/jmor.10474) 17051544

[5] Nemoto T. 1970 Feeding pattern of baleen whales in the ocean. In Marine food chains, pp. 241-252. Berkeley, CA: University of California Press.

[6] Kawamura A. 1980 A review of food of balaenopterid whales. Sci. Rep. Whales Res. Inst **32**, 155-197.

[7] Deméré TA, Berta A. 2008 Skull anatomy of the Oligocene toothed mysticete *Aetioceus weltoni* (Mammalia; Cetacea): implications for mysticete evolution and functional anatomy. Zool. J. Linn. Soc. **154**, 308-352. (10.1111/j.1096-3642.2008.00414.x)

[8] Fitzgerald EMG. 2010 The morphology and systematics of *Mammalodon colliveri* (Cetacea: Mysticeti), a toothed mysticete from the Oligocene of Australia. Zool. J. Linn. Soc. **158**, 367-476. (10.1111/j.1096-3642.2009.00572.x)

[9] Marx FG, Fordyce RE. 2015 Baleen boom and bust: a synthesis of mysticete phylogeny, diversity and disparity. R. Soc. Open Sci. **2**, 140434. (10.1098/rsos.140434) 26064636 PMC4448876

[10] Geisler JH, Boessenecker RW, Brown M, Beatty BL. 2017 The origin of filter feeding in whales. Curr. Biol. **27**, 2036-2042. (10.1016/j.cub.2017.06.003) 28669761

[11] Hocking DP, Marx FG, Park T, Fitzgerald EM, Evans AR. 2017 A behavioural framework for the evolution of feeding in predatory aquatic mammals. Proc. R. Soc. B **284**, 20162750. (10.1098/rspb.2016.2750) PMC5360926 28250183

[12] Fordyce RE, Marx FG. 2018 Gigantism precedes filter feeding in baleen whale evolution. Curr. Biol. **28**, 1670-1676.e2. (10.1016/j.cub.2018.04.027) 29754903

[13] Peredo CM, Pyenson ND, Marshall CD, Uhen MD. 2018 Tooth loss precedes the origin of baleen in whales. Curr. Biol. **28**, 3992-4000.e2. (10.1016/j.cub.2018.10.047) 30503622

[14] Berta A, Lanzetti A. 2020 Feeding in marine mammals: an integration of evolution and ecology through time. Palaeontol. Electron. **23**, a40. (10.26879/951)

[15] Pivorunas A. 1979 The feeding mechanisms of baleen whales. Am. Sci. **67**, 432-440.

[16] Fordyce RE, de Muizon C. 2001 Evolutionary history of whales: a review. In Secondary adaptation of tetrapods to life in water (eds J-M Mazin, V de Buffrenil), pp. 169-234. München, Germany: Pfeil.

[17] Tsai CH, Fordyce RE. 2018 A new archaic baleen whale *Toipahautea waitaki* (early Late Oligocene, New Zealand) and the origins of crown Mysticeti. R. Soc. Open Sci. **5**, 172453. (10.1098/rsos.172453) 29765689 PMC5936954

[18] Beatty B, Dooley A. 2009 Injuries in a mysticete skeleton from the Miocene of Virginia, with a discussion of buoyancy and the primitive feeding mode in the Chaeomysticeti. Jeffersoniana **20**, 1-28.

[19] Boessenecker RW, Fordyce RE. 2015 Anatomy, feeding ecology, and ontogeny of a transitional baleen whale: a new genus and species of Eomysticetidae (Mammalia: Cetacea) from the Oligocene of New Zealand. PeerJ **3**, e1129. (10.7717/peerj.1129) 26380800 PMC4570844

[20] Watkins WA, Schevill WE. 1979 Aerial observation of feeding behavior in four baleen whales: *Eubalaena glacialis, Balaenoptera borealis, Megaptera novaeangliae, and Balaenoptera physalus*. J. Mammal. **60**, 155-163. (10.2307/1379766)

[21] Marx FG, Kohno N. 2016 A new Miocene baleen whale from the Peruvian desert. R. Soc. Open Sci. **3**, 160542. (10.1098/rsos.160542) 27853573 PMC5098998

[22] Tsai CH, Fordyce RE. 2014 Disparate heterochronic processes in baleen whale evolution. Evol. Biol. **41**, 299-307. (10.1007/s11692-014-9269-4)

[23] Kimura T. 2002 Feeding strategy of an Early Miocene cetothere from the Toyama and Akeyo Formations, central Japan. Paleontol. Res. **6**, 179-189.

[24] Kimura T. 2005 Evolution of feeding strategies in the Mysticeti. Kaseki **77**, 14-21. [In Japanese with English abstract.]

[25] Bisconti M, Varola A. 2006 The oldest eschrichtiid mysticete and a new morphological diagnosis of Eschrichtiidae (gray whales). Rivista Italiana di Paleontologia e Stratigrafia (Research In Paleontology and Stratigraphy) **112**, 447-457.

[26] Werth AJ. 2006 Mandibular and dental variation and the evolution of suction feeding in Odontoceti. J. Mammal. **87**, 579-588. (10.1644/05-MAMM-A-279R1.1)

[27] Adli JJE, Deméré TA, Boessenecker RW. 2014 *Herpetocetus morrowi* (Cetacea: Mysticeti), a new species of diminutive baleen whale from the Upper Pliocene (Piacenzian) of California, USA, with observations on the evolution and relationships of the Cetotheriidae. Zool. J. Linn. Soc. **170**, 400-466. (10.1111/zoj.12108)

[28] Tarasenko KK. 2014 New genera of baleen whales (Cetacea, Mammalia) from the Miocene of the northern Caucasus and Ciscaucasia: 3. Zygiocetus gen. nov. (Middle Sarmatian, Adygea). Paleontol. J. **48**, 551-562. (10.1134/S0031030114050116)

[29] Hampe O, Hairapetian V, Mirzaie Ataabadi M, Orak Z. 2019 Preliminary report on a late Tortonian/Messinian balaenopterid cetacean (Mammalia, Mysticeti) from Sistan and Baluchestan Province (Iran). Geopersia **9**, 65-79.

[30] Bisconti M, Damarco P, Pavia M, Sorce B, Carnevale G. 2021 *Marzanoptera tersillae*, a new balaenopterid genus and species from the Pliocene of Piedmont, north-west Italy. Zool. J. Linn. Soc. **192**, 1253-1292. (10.1093/zoolinnean/zlaa131)

[31] Werth AJ. 2000 Feeding in marine mammals. In Feeding: form, function and evolution in tetrapod vertebrates, pp. 475-514. San Diego, CA: Academic Press.

[32] Mead JG, Fordyce RE. 2009 The therian skull: a lexicon with emphasis on the odontocetes. Smithson Contrib. Zool. **627**, 1-248. (10.5479/si.00810282.627)

[33] Rohlf FJ. 2015 The tps series of software. Hystrix **26**, 1-4.

[34] Klingenberg CP. 2011 MorphoJ: an integrated software package for geometric morphometrics. Mol. Ecol. Resour. **11**, 353-357. (10.1111/j.1755-0998.2010.02924.x) 21429143

[35] Rosel PE, Wilcox LA, Yamada TK, Mullin KD. 2021 A new species of baleen whale (*Balaenoptera*) from the Gulf of Mexico, with a review of its geographic distribution. Mar. Mamm. Sci. **37**, 577-610. (10.1111/mms.12776)

[36] Soldevilla MS, Hildebrand JA, Frasier KE, Dias LA, Martinez A, Mullin KD, Rosel PE, Garrison LP. 2017 Spatial distribution and dive behavior of Gulf of Mexico Bryde's whales: potential risk of vessel strikes and fisheries interactions. Endanger. Species Res. **32**, 533-550. (10.3354/esr00834)

[37] Nikaido M, Hamilton H, Makino H, Sasaki T, Takahashi K, Goto M, Kanda N, Pastene LA, Okada N. 2006 Baleen whale phylogeny and a past extensive radiation event revealed by SINE insertion analysis. Mol. Biol. Evol. **23**, 866-873. (10.1093/molbev/msj071) 16330660

[38] Sasaki T, Nikaido M, Wada S, Yamada TK, Cao Y, Hasegawa M, Okada N. 2006 *Balaenoptera omurai* is a newly discovered baleen whale that represents an ancient evolutionary lineage. Mol. Phylogenet. Evol. **41**, 40-52. (10.1016/j.ympev.2006.03.032) 16843687

[39] McGowen MR, Spaulding M, Gatesy J. 2009 Divergence date estimation and a comprehensive molecular tree of extant cetaceans. Mol. Phylogenet. Evol. **53**, 891-906. (10.1016/j.ympev.2009.08.018) 19699809

[40] Steeman ME et al. 2009 Radiation of extant cetaceans driven by restructuring of the oceans. Syst. Biol. **58**, 573-585. (10.1093/sysbio/syp060) 20525610 PMC2777972

[41] Buono MR, Fernández MS, Cozzuol MA, Cuitiño JI, Fitzgerald EMG. 2017 The early Miocene balaenid *Morenocetus parvus* from Patagonia (Argentina) and the evolution of right whales. PeerJ **5**, e4148. (10.7717/peerj.4148) 29302389 PMC5742523

[42] Lambert O, Martínez-Cáceres M, Bianucci G, Di Celma C, Salas-Gismondi R, Steurbaut E, Urbina M, de Muizon C. 2017 Earliest mysticete from the Late Eocene of Peru sheds new light on the origin of baleen whales. Curr. Biol. **27**, 1535-1541. (10.1016/j.cub.2017.04.026) 28502655

[43] Gol'din P. 2018 New Paratethyan dwarf baleen whales mark the origin of cetotheres. PeerJ **6**, e5800. (10.7717/peerj.5800) 30356949 PMC6193469

[44] De Muizon C, Bianucci G, Martinez-Caceres M, Lambert O. 2019 *Mystacodon selenensis*, the earliest known toothed mysticete (Cetacea, Mammalia) from the late Eocene of Peru: anatomy, phylogeny, and feeding adaptations. Geodiversitas **41**, 401-499. (10.5252/geodiversitas2019v41a11)

[45] Marx FG, Post K, Bosselaers M, Munsterman DK. 2019 A large Late Miocene cetotheriid (Cetacea, Mysticeti) from the Netherlands clarifies the status of Tranatocetidae. PeerJ **7**, e6426. (10.7717/peerj.6426) 30783574 PMC6377596

[46] de Lavigerie GD, Bosselaers M, Goolaerts S, Park T, Lambert O, Marx FG. 2020 New Pliocene right whale from Belgium informs balaenid phylogeny and function. J. Syst. Paleontol. **18**, 1141-1166. (10.1080/14772019.2020.1746422)

[47] Kimura T, Hasegawa Y. 2021 Second report on the new material of *Joumocetus shimizui* from the Miocene Haraichi Formation, Annaka Group, Gunma, Japan. Bullet. Gunma Mus. Nat. Hist. **25**, 59-64.

[48] Geisler JH, McGowen MR, Yang G, Gatesy J. 2011 A supermatrix analysis of genomic, morphological, and paleontological data from crown Cetacea. BMC Evol. Biol. **11**, 112. (10.1186/1471-2148-11-112) 21518443 PMC3114740

[49] Okazaki Y. 2012 A new mysticete from the upper Oligocene Ashiya Group, Kyushu, Japan and its significance to mysticete evolution. Bullet. Kitakyushu Mus. Nat. Hist. Hum. Hist. Ser. A (Natural History) **10**, 129-152.

[50] Sanders AE, Barnes LG. 2002 Paleontology of the late Oligocene Ashley and Chandler Bridge formations of South Carolina, 3: Eomysticetidae, a new family of primitive mysticetes (Mammalia: Cetacea). Smithsonian Contrib. Paleobiol. **93**, 313-356.

[51] Tsai CH, Fordyce RE. 2015 The earliest gulp-feeding mysticete (Cetacea: Mysticeti) from the Oligocene of New Zealand. J. Mamm. Evol. **22**, 1-26. (10.1007/s10914-014-9263-8)

[52] Bouetel V. 2005 Phylogenetic implications of skull structure and feeding behavior in balaenopterids (Cetacea, Mysticeti). J. Mammal. **86**, 139-146. (10.1644/1545-1542(2005)086<0139:PIOSSA>2.0.CO;2)

[53] Churchill M, Berta A, Deméré T. 2012 The systematics of right whales (Mysticeti: Balaenidae). Mar. Mamm. Sci. **28**, 497-521. (10.1111/j.1748-7692.2011.00504.x)

[54] Goldbogen J, Cade D, Calambokidis J, Friedlaender A, Potvin J, Segre P, Werth A. 2017 How baleen whales feed: the biomechanics of engulfment and filtration. Annu. Rev. Mar. Sci. **9**, 367-386. (10.1146/annurev-marine-122414-033905) 27620830

[55] Werth AJ. 2004 Models of hydrodynamic flow in the bowhead whale filter feeding apparatus. J. Exp. Biol. **207**, 3569-3580. (10.1242/jeb.01202) 15339953

[56] Werth AJ, Potvin J. 2016 Baleen hydrodynamics and morphology of cross-flow filtration in balaenid whale suspension feeding. PLoS ONE **11**, e0150106. (10.1371/journal.pone.0150106) 26918630 PMC4769178

[57] Brainerd EL. 2001 Caught in the crossflow. Nature **412**, 387-388. (10.1038/35086666) 11473292

[58] Bisconti M. 2012 Comparative osteology and phylogenetic relationships of *Miocaperea pulchra*, the first fossil pygmy right whale genus and species (Cetacea, Mysticeti, Neobalaenidae). Zool. J. Linn. Soc. **166**, 876-911. (10.1111/j.1096-3642.2012.00862.x)

[59] Bisconti M. 2005 Skull morphology and phylogenetic relationships of a new diminutive balaenid from the Lower Pliocene of Belgium. Palaeontology **48**, 793-816. (10.1111/j.1475-4983.2005.00488.x)

[60] Bisconti M. 2003 Evolutionary history of Balaenidae. Cranium **20**, 9-50.

[61] Gol'din P, Startsev D, Krakhmalnaya T. 2014 The anatomy of *Cetotherium riabinini* Hofstein, 1948, a baleen whale from the late Miocene of Ukraine. Acta Palaeontol. Pol. **59**, 795-814.

[62] Tanaha Y. 2022 Rostrum morphology and feeding strategy of the baleen whale indicate that right whales and pygmy right whales became skimmers independently. Figshare. (10.6084/m9.figshare.c.6296311) PMC9682309 36425522

