# Correction to: ‘Rostrum morphology and feeding strategy of the baleen whale indicate that right whales and pygmy right whales became skimmers independently’ (2022), by Tanaka

**DOI:** 10.1098/rsos.241686

**Published:** 2024-10-23

**Authors:** Yoshihiro Tanaka

**Affiliations:** ^1^ Sapporo Museum Activity Center, Sapporo, Hokkaido, Japan

**Keywords:** lunge feeding, skim feeding, cetacea, mammalia, convergent

*R. Soc. Open Sci.* 9, 221353 (Published online 23 November 2022). ( https://doi.org/10.1098/rsos.221353).

In the article ‘Rostrum morphology and feeding strategy of the baleen whale indicate that right whales and pygmy right whales became skimmers independently’ by Yoshihiro Tanaka [1], the author wants to make the following corrections to the paper.

Figure 6, explanations of *Balaenella brachyrhynu*: ‘multiple prey capture strategy’ should read ‘transitional feeding strategy from a primitive one to skim feeding’.

The author apologizes for not correcting the errors in the proof.


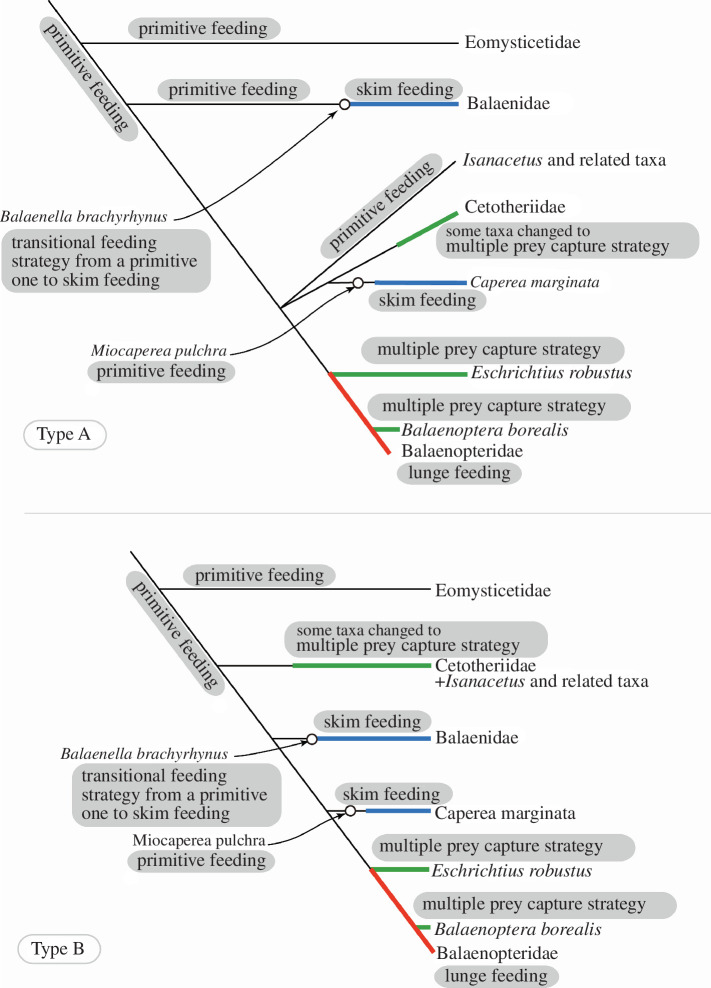


Figure 6. Feeding strategy evolution among the Chaeomysticeti: true baleen whales with two types of phylogenetic hypotheses. Thin green lines indicate linages with unknown primitive feedings. Thick lines represent shifts from primitive feeding to skim feeding in blue, to multiple prey capture strategy in green and lunge feeding as seen among modern balaenopterids in red.
